# Defense response of strawberry plants against *Botrytis cinerea* influenced by coriander extract and essential oil

**DOI:** 10.3389/fpls.2022.1098048

**Published:** 2023-01-05

**Authors:** Lina Dėnė, Kristina Laužikė, Neringa Rasiukevičiūtė, Simona Chrapačienė, Aušra Brazaitytė, Akvilė Viršilė, Viktorija Vaštakaitė-Kairienė, Jurga Miliauskienė, Rūta Sutulienė, Giedrė Samuolienė, Alma Valiuškaitė

**Affiliations:** ^1^ Laboratory of Plant Protection, Institute of Horticulture, Lithuanian Research Centre for Agriculture and Forestry, Babtai, Lithuania; ^2^ Laboratory of Plant Physiology, Institute of Horticulture, Lithuanian Research Centre for Agriculture and Forestry, Babtai, Lithuania

**Keywords:** biocontrol, *coriandrum sativum*, grey mold, disease severity, antioxidant activity, photosynthesis, sustainable plant protection

## Abstract

Essential oils and extracts are investigated in sustainable plant protection area lately. Alternative antifungal substances are especially relevant for major economic-relevance pathogens, like *Botrytis cinerea* (causal agent of strawberry grey mold), control. However, the reaction of plants to alternative protection with plant-origin products is currently unknown. Induced stress in plants causes changes in antioxidant and photosynthetic systems. The aim of the research was to determine the defense response of strawberry plants under application of coriander seed products. In the first step of the research, we determined coriander seed (*Coriandrum sativum*), black seed (Nigella sativa) and peppermint leaf (*Menta* × *piperita*) products’ antifungal activity against *B. cinerea in vitro*. Secondly, we continued evaluation of antifungal activity under controlled environment on strawberry plants of the most effective coriander seed products. Additionally, we evaluated the antioxidant and photosynthetic parameters in strawberries, to examine the response of plants. Antifungal activity on strawberries was determined based on grey mold incidence and severity after application of coriander products. Impact on photosynthetic system was examined measuring photosynthetic rate, transpiration rate, stomatal conductance, and intercellular to ambient CO_2_ concentration. Strawberry leaves were collected at the end of the experiment to analyze the antioxidant response. The highest antifungal activity both *in vitro* and on strawberries had coriander seed essential oil, which decreased grey mold severity. Coriander extract increased the photosynthetic capacity and antioxidant response of strawberry plants, however had negative effect on suppression of grey mold. In most cases, the essential oil activated antioxidant response of strawberry plants lower than extract. Our study results provide no direct impact of increased photosynthetic capacity values and antifungal effect after treatment with natural oils. The highest concentrations of coriander essential oil and extract potentially demonstrated a phytotoxic effect.

## 1 Introduction

Healthy fruit-bearing plants and high-quality yield are prioritized in the horticultural market. Currently, in agriculture, a lower usage of chemical pesticides for disease management should be achieved to reduce existing and occurring consequences to the environment and food safety (Nxumalo et al., 2021). Therefore, attention is paid to the investigation of the effectiveness of naturally occurring pesticides from plants against pathogens (Raveau et al., 2020; Soleha et al., 2022). The plant’s defense response is a complex system depending on various elements. Despite decreasing damage to pathogens, natural compounds can also have a growth-enhancing effect (Andresen and Cedergreen, 2010), affect photosynthetic capacity (Yao et al., 2020), and phenolic compounds (Karlund et al., 2014; Wei et al., 2018). An additional value would be provided to the natural compounds by registering not only the antifungal effect but also the impact on other plant factors.

Previous research on antifungal activity shows that there is a demand for non-chemical measures for control of plant pathogens (Zaker and Mosallanejad, 2010; Mohammadi et al., 2014; Zabka et al., 2014; Ikeura and Koabayashi, 2015; Zatla et al., 2017; Moghadam et al., 2019). Coriander leaves and stem extracts showed high antifungal activity against *Botryotinia fuckeliana*, *Glomerella cingulata*, *Fusarium oxysporum*, and *Pectobacterium carotovorum* subsp. *carotovorum* (Ikeura and Koabayashi, 2015). The essential oil of coriander had an inhibitory effect on *Aspergillus flavus* (Lasram et al., 2019), and *Leucoagaricus gongylophorus* (Morais et al., 2015). Besides, it demonstrated antimicrobial properties for food preservation (Zamindar et al., 2016; El-Sayed et al., 2022), while the anti-yeast effect of the black seed extract was observed (Nadaf et al., 2015). Although there are studies on the effectiveness of coriander, peppermint, and black seed extracts *in vitro* against fungal pathogens, only a few studies on *Botrytis cinerea* under *in vitro* conditions could be found (Mohammadi et al., 2014). Additionally, the comparison of different forms of natural compounds from plants is not widely investigated as well as the effectiveness of the extracts *in vivo*.

Based on the literature mentioned above, the oils and extracts of coriander, peppermint, and black seed can potentially have antifungal activity against *B. cinerea* and become a biopesticide for strawberry grey mold. Also, these products demonstrated antioxidant activity (Wangensteen et al., 2004; Zheljazkov et al., 2014). Phenolic compounds and antioxidant activity were mainly investigated in berries but not in the plant itself (Wei et al., 2018; Abd-Elkader et al., 2021). The content of phenolic compounds is one of the main indicators of the response of plants to stress (Elstner et al., 1993; Rivero et al., 2001) and indicate plants natural defense against pathogen (Karlund et al., 2014). Studies show fruits’ total phenolics and antioxidant activity are highly responsive to *B. cinerea* (Bui et al., 2019; Tzortzakis, 2019). Nevertheless, a preharvest antioxidant response in strawberry plants after treatment with extracts and essential oils was not investigated.

An important primary metabolic process for plants is photosynthesis. It provides energy and material for plant growth and is responsible for plant defense mechanisms. One of the stomata’s vital roles in plants is to regulate gas exchange. However, its channels may also be favorable for pathogen entry (Francesconi and Balestra, 2020). Reducing stomata size (to defend from pathogens) affects gas exchange that leads to inhibition in CO_2_ assimilation and photosynthetic rate (Ahammed et al., 2021; Geng et al., 2022). Pathogens induce stress in strawberry plants, leading to elevated antioxidant activities (Petrasch et al., 2019; Sekmen Cetinel et al., 2021). In addition, with the help of biological control agents, secondary metabolites in their synthesis and metabolic pathways enhance resistance to stress, biotic and abiotic (Ferreira and Musumeci, 2021). Although there are reports on the antifungal activity of extracts and essential oils and their antioxidant activity itself, so far there is no complex research conducting antifungal activity evaluation together with the impact on the antioxidant system of plants.

Therefore, the aim of the study was to determine the defense response of strawberry plants under application of coriander seed products. To obtain this, firstly, we selected the extract from coriander seed (*Coriandrum sativum*), black seed (Nigella sativa) and peppermint leaf (*Menta* × *piperita*) extracts based on the highest antifungal activity against *B. cinerea in vitro*. Secondly, we examined the possibilities of strawberry *B. cinerea* control under controlled environment on strawberries using three different forms (extract, essential oil, and hydrosol) of selected extract (*C. sativum*) and determined strawberry antioxidant and physiological response.

## 2 Materials and methods

### 2.1 Selection of the plant extract *in vitro*

In this part of study, natural products were prepared and investigated for antifungal activity against *B. cinerea in vitro*. Further, the most effective extract was prepared in three different forms and investigated for minimal inhibitory concentrations (MIC), which were used in the experiments with strawberry plants.

#### 2.1.1 Natural products preparation

Coriander seed (*Coriandrum sativum*), peppermint leaf (*Menta* × *piperita*), and black seed (*Nigella sativa*) CO_2_ extracts were used for the first part of the evaluation of the antifungal effect against *Botrytis cinerea in vitro* at different concentrations. Coriander (*Coriandrum sativum*) essential oil, extract, and hydrosol were used for the second part of the evaluation of the antifungal effect *in vitro* and on strawberry plants under controlled environment.

Dried plant material: coriander seeds, peppermint leaves, and black seeds were obtained from Origanum (Lithuania). Extracts were produced by the previously published method and parameters of subcritical CO_2_ extraction (Šernaitė et al., 2020). Meanwhile, essential oil and hydrosol were obtained by hydrodistillation with Clevenger apparatus.

#### 2.1.2 Antifungal activity *in vitro*

The first part of the experiment *in vitro* on the antifungal effect of the extracts was carried out by mixing coriander, peppermint, and black seed extracts with potato dextrose agar (PDA) at 0.02-0.20% concentrations and pouring them into Petri dishes. Each Petri dish was inoculated with a 7-day old 7 mm diameter mycelium disc of *B. cinerea*. The experiment was kept at 22 ± 2^°^C in the dark for 7 days. The antifungal effect was evaluated by measuring the radial colony growth of the pathogen at 2, 4, and 7 days after inoculation (DAI). The extract, causing the lowest radial colony growth of *B. cinerea* at investigated concentrations, was considered as the most effective and was used for the determination of MIC.

#### 2.1.3 Determination of minimal inhibitory concentration

In the second part of the experiment *in vitro*, three different forms of the most effective coriander extract were used. Extract, essential oil, and hydrosol of coriander seed were separately mixed with PDA at 0.06-0.18% concentrations. Inoculation and evaluation were made as described in the first part of *in vitro* experiments. We considered MIC as the lowest concentration with the highest antifungal activity, and MIC of the essential oil as the lowest concentration with less than 1 cm radial colony growth of *B. cinerea* at 5 DAI. These concentrations were used as a base for the applications on strawberry plants.

### 2.2 Antifungal activity on strawberry plants under controlled environment

Strawberries (cv. Deluxe) were planted, in the 15x15 cm plastic pots with 1:1 black soil and peat soil for vegetable mix (Durpeta, Lithuania) in greenhouse, under supplemental high-pressure sodium lamps lighting (200 μmol m^-2^ s^-1^), under 16 h photoperiod. Plants were watered as needed and fertilized every week from 1.5 months after planting, with N 34 (10 g/L) and NPK 14-10-25 (10 g/L) (Baltic Agro). Strawberries were transferred into a controlled environment at 21°C temperature, and 70% humidity 2 months after planting. The acclimatization lasted for 2 days.

24h before the inoculation, the humidity was increased to 100% and plants were covered under agro-film to make suitable conditions for the infection of grey mold. After 24h, spraying mixtures with three coriander seed products were prepared by mixing them with sterilized distilled water at 0.12-1.6% concentrations and 1% Tween 80 as a surfactant (**Table 1**). Hydrosol and sterilized distilled water mixture (1:1) was used instead of only water in two spraying mixtures (Ext 0.16+h, Eo 0.12+h). Inoculated control (Control inoc) was sprayed only with sterile distilled water. Additionally, non-inoculated and not sprayed control treatment was used in the experiment for comparison (Control 0).

**Table 1:** Spraying mixtures for strawberry application under controlled environment.

Treatment	Abbreviation
Control not inoculated	Control 0
Control inoculated	Control inoc
Coriander extract 0.16%	Ext 0.16
Coriander essential oil 0.12%	Eo 0.12
Coriander extract 0.16% + hydrosol	Ext 0.16+h
Coriander essential oil 0.12% + hydrosol	Eo 0.12+h
Coriander extract 1.6%	Ext 1.6
Coriander essential oil 1.2%	Eo 1.2

The experiment was arranged in randomized blocks, 3 replicates (five plants) per treatment. 5 ml of prepared mixtures were sprayed on each treatment. Three wounds were made on three different middle leaves of each strawberry trifoliate. 9 mm mycelium plug of *B. cinerea* was put on each wound. Strawberries were covered under agro-film again and kept at 21°C and 100% humidity for 48h. After 48h, agro-film was removed.

**Figure 1:**
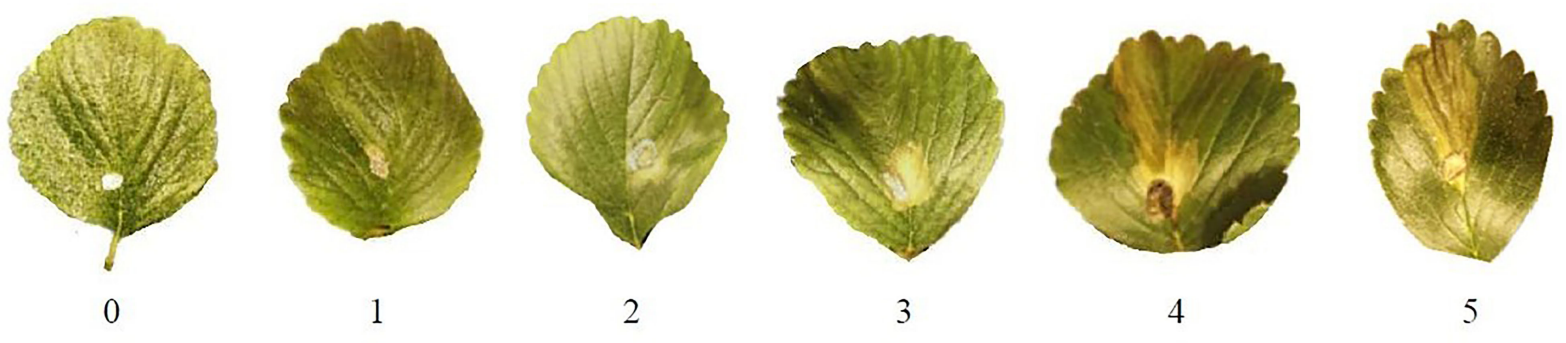
Grey mold severity scale on strawberry leaves suggested by the authors.


The experiment was evaluated daily, and the first grey mold incidence and severity registered when the first infection symptoms were observed. For each replicate, disease severity in inoculated leaves area firstly was evaluated with scores from 0 to 5, where: 0 – no infection; 1 -<5%; 2 -<10%; 3 -<15%; 4< 20% and 5 – >21% infected (**Figure 1**), and then scores were used to calculate percentage value using formula (Dabkevičius and Brazauskienė, 2007):

$$Disease\;severity(\%)=\frac{\Sigma (B\cdot a)}{AK}\times 100$$

where: A – number of inspected leaves, B – number of leaves damaged by grey mold, a – number of leaves with the same damage score, K – the highest score of the scale, Σ – the sum of the products of the number of leaves with the same damage score and the score values.

Disease incidence was calculated as the proportion of infected leaves. All evaluations were made 3, 6, and 8 days after inoculation.

### 2.3 Determination of the antioxidant activity in strawberries

Extracts were prepared by grinding 0,5 g of strawberry leaves with liquid nitrogen and diluting with 5 mL of 80% methanol. Each of three biological replicates consisted of at least five conjugated plants leaves and were repeated in three analytical replicates. Further, biochemical analyses were performed from the prepared extract. The antioxidant assay was carried out as in Perez-Lopez et al. (2014), using the radical cation ABTS (2,2’-azino-di[3-ethylbenzthiazoline sulphonate]) generated following Re et al. (1999). The activity was assayed after the addition of 10 μl of the extract to the radical solution, previously diluted to reach an absorbance value of 0.70 ± 0.05 at 734 nm. After 10 min of reaction, the decrease in absorbance was compared to that caused by Trolox standard solutions in the range 0.2–1.5 mM. The activity was expressed as μmol Trolox equivalent antioxidant capacity (TEAC) g−1 dry weight (DW) of plant material.

The antioxidant activity (by DPPH) of methanol extracts of the investigated plants was evaluated spectrophotometrically relating to the 2,2–diphenyl–1–picrylhydrazyl (DPPH•) free radical scavenging capacity (Ragaee et al., 2006). The absorbance scanned after 16 minutes from the beginning of the reaction at 515 nm was used for the calculation of the ability of seed material to scavenge DPPH• free radicals (μmol g-1).

Total phenols in methanolic extracts were determined as previously reported by Mazzoncini et al. (2015) following the reaction at 735 nm between phenols and the Folin-Ciocalteu reagent. Gallic acid was used as a standard to prepare a calibration curve to quantify phenolics amounts. Total phenols were expressed as gallic acid equivalents (GAE) g−1 DW.

### 2.4 Determination of the photosynthetic indices

Photosynthetic rate (Pr, μmol CO2 m^−2^ s^−1^), transpiration rate (Tr, mmol H2O m^−2^ s^−1^), stomatal conductance (g_s_, mol H_2_O m^−2^ s^−1^), intercellular to ambient CO_2_ concentration (Ci/Ca) was determined 9:00-12:00 am by using an LI-6400XT portable open flow gas exchange system (Li-COR 6400XT Biosciences, Lincoln, USA). Fully developed healthy leaves near the inoculated leaf from the plant, were chosen for measurements, five plants were measured for one minute. Reference air (CO_2_ 400 μmol mol^-1^), light intensity (1000 μmol m^-2^ s^-1^), and the flow rate of gas pump (500 mmol s^-1^) were set.

### 2.5 Statistical analysis

Statistical analysis of antifungal activity data was made using SAS Enterprise Guide 7.1 program (SAS Inc., USA). One-way analysis of variance (ANOVA) procedure was performed. Means of three replicates (n = 3) were compared by Duncan’s multiple range test (p< 0.05). Statistical analysis for plant physiology (photosynthetic and antioxidant systems response) data was performed using Microsoft Excel 2016 and Addinsoft XLSTAT 2022.1 XLSTAT statistical and data analysis (Long Island, NY, USA). The data are presented as means of three replicates (n = 3) linked to the sampling points. One-way (ANOVA) followed by Tukey’s significant difference test (p< 0.05) for multiple comparisons was used to evaluate differences between means of measurements. Multivariate principal component analysis (PCA) was performed to determine the statistical relations between natural product applications, used in the experiment. The results are presented in a three PCA scatterplot that indicates distinct levels of: 1. combined extracts antifungal activity and treated strawberry plants physiological data; 2. only treated strawberry plants physiological data; 3. only extracts antifungal activity data.

## 3 Results

### 3.1 Selection of the plant extract *in vitro*

**Figure 2:**
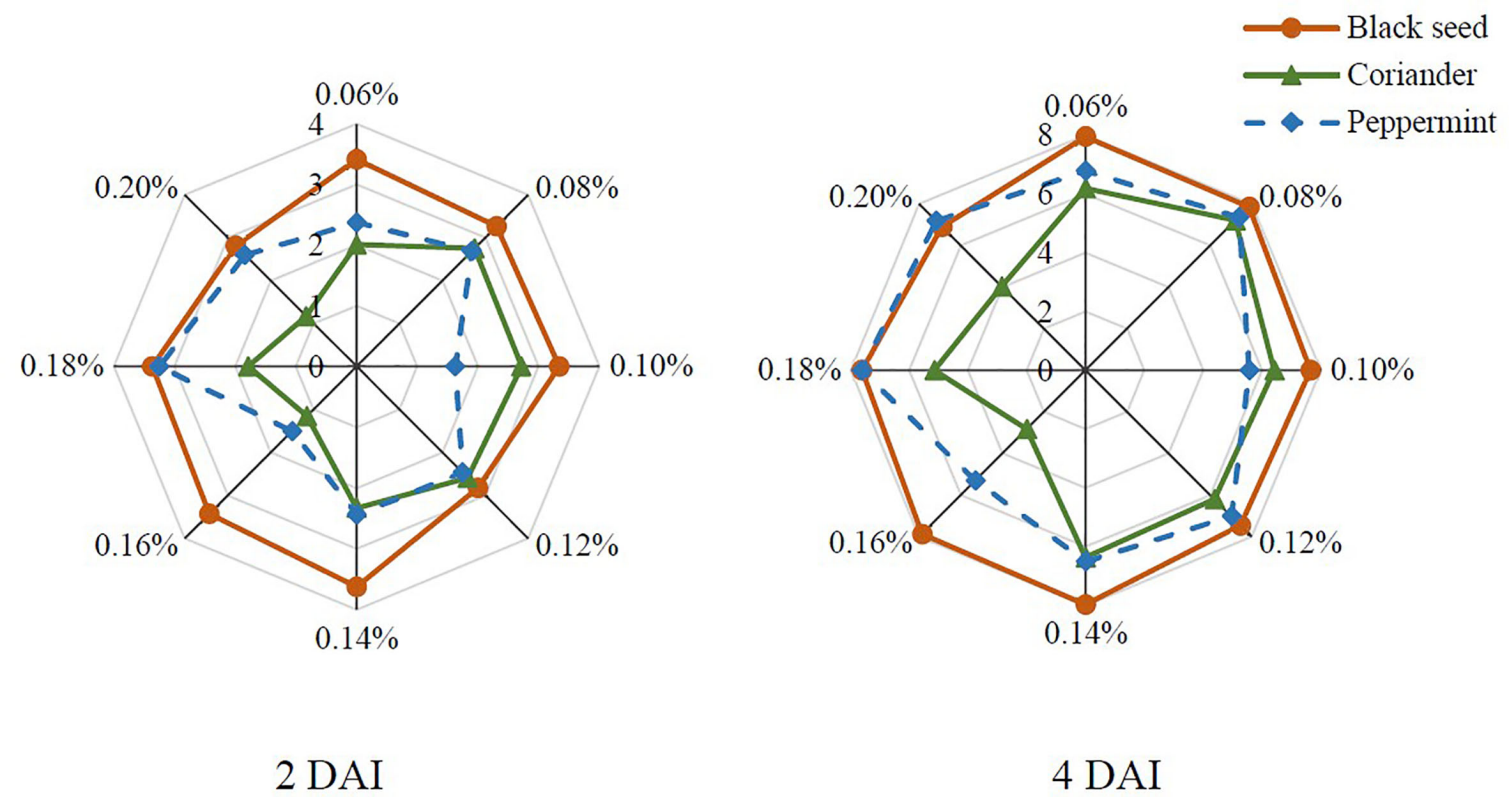
The radial colony growth (cm) of *B. cinerea* on the PDA with black seed (*Nigella sativa*), coriander (*Coriandrum sativum*), and peppermint (*Menta* × *piperita*) extracts at different concentrations (%); DAI – days after inoculation.

Three plant extracts were evaluated for antifungal activity against *B. cinerea in vitro*. The radial colony growth of *B. cinerea* under the action of black seed, coriander, and peppermint extracts is presented in **Figure 2**. The use of 0.16% of coriander and peppermint extracts and 0.20% of coriander extract resulted in the lowest growth of the pathogen at 2 DAI. Coriander extract remained with the highest antifungal activity at 0.16-0.20% concentration range at 4 DAI. The most effective concentration was 0.16%. Peppermint extract lost efficiency at 4 DAI. The lowest antifungal activity was observed with blackseed extract at both DAI.

As coriander extract was observed as the most effective against *B. cinerea*, *in vitro* study was continued with three different products from coriander seed. Determined MIC (**Table 2**) were used for strawberry treatment under controlled environment. Coriander essential oil showed the lowest MIC. Meanwhile, *B. cinerea* was not inhibited by any investigated concentration of hydrosol.

**Table 2:** Minimal inhibitory concentrations (MIC) of products from coriander seed.

Product type	2 DAI	5 DAI
Extract	0.16%	0.16%
Essential oil	0.06%	0.12%
Hydrosol	>0.20%	>0.20%

### 3.2 Antifungal activity of coriander seed products under controlled environment

**Figure 3:**
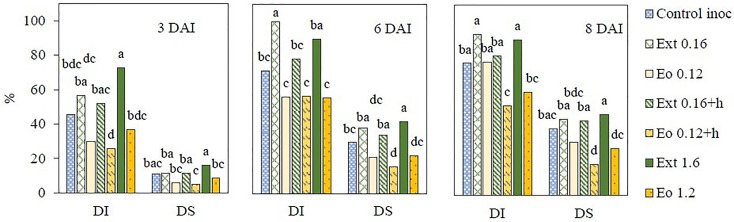
Grey mould incidence (DI, %) and severity (DS, %) on strawberry plants after application of coriander seed products; DAI – days after inoculation. Different letters indicate significant differences between the means of the treatments (Duncan’s multiple range test, *p* < 0.05).

Disease incidence and severity were evaluated to investigate the antifungal activity of natural products from coriander seed against strawberry grey mold induced by *B. cinerea*. The lowest disease incidence and severity were observed in essential oil treatments Eo 0.12 and Eo 0.12+h at 3 and 6 DAI (**Figure 3**). Eo 0.12+h and Eo 1.2 treated strawberries had the lowest grey mold parameters at 8 DAI. Treatments with extract (Extr1, Extr1+h, Extr2) were not effective in reducing the infection and had higher disease incidence and severity than control treatment at all DAI. Ext 0.16+h demonstrated the best results compared to the two other extract applications.

### 3.3 Antioxidant compounds in strawberries after application with coriander seed products

The radical scavenging activity and total phenolic compounds (TPC) in strawberry plants at 8 DAI are shown in **Table 3**. Inoculation increased strawberry’s antioxidant system activity up to two times, as well as total phenol content. The highest DPPH, ABTS and TPC were determined in Ext 1.6 and Eo 1.2 treatments. Lower than inoculated control DPPH and ABTS scavenging activity was observed in other treatments except for Ext 0.16+h, which was almost equal to control at ABTS. The highest total phenolic compounds were in Ext 1.6 and Eo 1.2 treatments. Meanwhile, Eo 0.12, Ext 0.16+h and Eo 0.12+h treatments resulted in lower than control treatment TPC.

**Table 3:** Effect of coriander seed products applications on antioxidant system response of strawberry leaves.

Treatment	DPPH, mM TE/g	ABTS, mM TE/g	TPC, mg/g
Control 0	123.90 ± 5.00 b	132.20 ± 5.06 c	10.31 ± 0.22 d
Control inoc	178.70 ± 35.24 a	284.37 ± 25.76 a	25.98 ± 1.13 ab
Ext 0.16	168.02 ± 13.46 ab	239.60 ± 30.21 ab	23.80 ± 3.64 ab
Eo 0.12	155.31 ± 23.29 ab	254.45 ± 41.53 a	20.23 ± 4.10 bc
Ext 0.16+h	180.23 ± 6.61 a	265.53 ± 11.21 a	20.20 ± 0.85 bc
Eo 0.12+h	154.69 ± 17.32 ab	164.23 ± 22.35 bc	14.46 ± 1.99 cd
Ext 1.6	197.66 ± 10.97 a	279.12 ± 24.84 a	28.38 ± 4.07 a
Eo 1.2	206.30 ± 24.45 a	291.50 ± 45.63 a	25.82 ± 2.36 ab

Values are mean ± SE of 3 replicates. Different letters indicate significant differences between the means of the treatments (Tukey’s significant difference test, p < 0.05).

### 3.4 Photosynthetic response of strawberry leaves after application with coriander seed products

Inoculation decreased the photosynthetic rate of strawberries, but increased transpiration compared to not inoculated strawberries in the majority of applications (**Table 4**). All the applications significantly increased photosynthetic rate except Eo 0.12 and Eo 1.2. Eo 1.2 significantly decreased the photosynthetic rate of strawberry leaves. Meanwhile, Eo 0.12 effect was not significant compared to the control. The Eo 1.2 effect was manifested by the closing of stomata, which caused a drop in transpiration and the photosynthetic rate (**Table 4**). Ext 0.16 and Ext 0.16+h opened the stomata and increased stomatal conductance up to two times compared to both control treatments. Transpiration increased significantly 26.6 and 48.5% in treatments, respectively, compared to the not inoculated plants. Ext 1.6 and Eo 1.2 decreased intercellular CO_2_ mostly compared to inoculated plants.

**Table 4:** Photosynthetic capacity of strawberry leaves: Photosynthetic rate (P_n_), Stomatal conductance g_s_, transpiration rate (E), intercellular CO_2_ (Ci/Ca).

Treatment	P_n_ µmol CO_2_ m^−2^ s^−1^	g_s_ mol H_2_O m^−2^ s^−1^	E mmol H_2_O ·m^−2^ · s^−1^	Ci/Ca
Control 0	10.31 ± 0.10 a	0.36 ± 0.01 cd	1.73 ± 0.01 d	330.39 ± 1.39 b
Control inoc	6.84 ± 0.06 c	0.35 ± 0.03 cd	2.22 ± 0.11 bc	352.07 ± 1.93 a
Ext 0.16	11.10 ± 0.26 a	0.56 ± 0.01 ab	2.57 ± 0.01 ab	348.09 ± 0.82 a
Eo 0.12	7.09 ± 2.29 c	0.31 ± 0.14 de	1.70 ± 0.49 d	347.51 ± 1.52 a
Ext 0.16+h	8.39 ± 0.88 b	0.64 ± 0.17 a	2.78 ± 0.35 a	359.21 ± 11.1 a
Eo 0.12+h	8.24 ± 0.23 b	0.49 ± 0.02 abc	2.05 ± 0.06 cd	354.79 ± 2.42 a
Ext 1.6	11.81 ± 0.04 a	0.44 ± 0.01 bcd	2.19 ± 0.04 c	333.21 ± 0.50 b
Eo 1.2	5.53 ± 0.05 d	0.16 ± 0.01 e	1.09 ± 0.04 e	330.77 ± 3.28 b

Values are mean ± SE of 3 replicates. Different letters indicate significant differences between the means of the treatments (Tukey’s significant difference test, p< 0.05).

### 3.5 Principal component analysis

**Figure 4:**
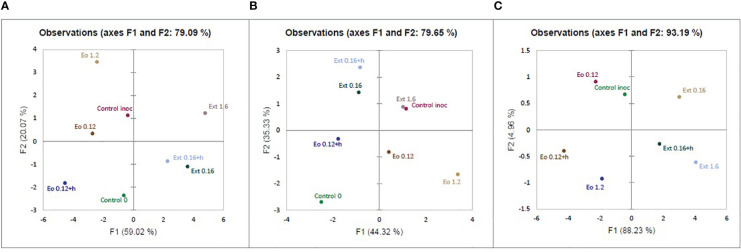
Principal component analysis (PCA) of: combined extracts antifungal activity and treated strawberry plants physiological data **(A)**; only treated strawberry plants physiological data **(B)**, and only extracts antifungal activity data **(C)**.

After evaluating the results of the experiment, the principal component analysis (PCA) covered 79.09% of all data and divided the used applications into separate groups (**Figure 4A**). The F1 axis covered 59.02% of the data and the applications according to the effect on strawberries were divided into two groups, with essential oils and control in one, and extracts in the other. The F2 axis (covering 20.07% of the data) separated the treatments by concentrations. Analyzing data only from treated strawberry plants’ physiological response, PCA covered 79.65% of all data. Strawberries’ physiological response is divided into similar groups: one extracts and inoculated control and the second one essential oil and not inoculated control (**Figure 4B**). Meanwhile, PCA data from antifungal activity aspects covered 93.13% data. According to antifungal activity data, essential oils and extracts were also divided into groups by effect on disease severity (**Figure 4C**).

## 4 Discussion

### 4.1 Antifungal activity of coriander seed products *in vitro* and on strawberry plants

The results of our study provide relevant information on the further step of natural oils application *in vivo* for grey mold prevention. In the first part of our study, we determined coriander CO_2_ extract as the most effective amongst other investigated extracts. Until now, coriander essential oil or extract were mostly investigated for antifungal activity *in vitro* (Zaker and Mosallanejad, 2010; Mohammadi et al., 2014; Ikeura and Koabayashi, 2015; Zatla et al., 2017; Moghadam et al., 2019). The high antifungal activity of coriander essential oil against *Alternaria alternata*, *Stachybotrys chartarum*, *Cladosporium cladosporioides*, *Aspergillus niger* fungi was stated in other study (Zabka et al., 2014), which agrees with our *in vitro* results. It revealed the differences in coriander oil antifungal activity, depending on the active components of the investigated distilled oil fraction (Delaquis et al., 2002). Through the time of our experiments with strawberries, coriander essential oil lowered grey mold severity *in vivo*. However, the opposite effect was observed after treatment with coriander extract. Such data emphasize the differences between the products obtained using different techniques. However, the lower concentration of coriander essential oil (0.12%) was grouped statistically with inoculated control according to their similar effect on disease severity. Moreover, we did not observe the positive effect of increased product concentration, as higher antifungal activity was registered after adding hydrosol than with a higher concentration. There is no data of application of plant extracts on strawberries under controlled climate conditions so far, thus our results provide valuable information for the further practical use of coriander extracts on horticultural crops.

### 4.2 Antioxidant response in strawberries after application with coriander seed products

Antioxidant capacity, both DPPH and ABTS, increased together with increased concentration. Active antioxidant system response after treatment with coriander extract was not efficient in decreasing disease severity and incidence. Evaluating disease incidence, a negative effect of coriander extract was observed, as more plants were found to be infected compared to an inoculated plant. Coriander essential oil activated antioxidant response of strawberry plant lower than extract (except with Eo 1.2 treatment). Reduced stress for the plant after inoculation may have resulted in decreased disease parameters. On the contrary, more observations should be performed with the highest used concentrations (Eo 1.2 and Ext 1.6 treatments) as both extract and essential oil increased strawberries’ total phenolic compounds and antioxidant capacity. Inoculation increases plants’ antioxidant system response. Speaking about the increase level, we could conclude that a slight increase of the antioxidant system was more useful in preventing grey mold infection than a higher increase. A similar effect was found with birch wood distillate, which increased the phenolics in strawberry leaves (Karlund et al., 2014). Phenolic compounds are described as responsible for antifungal activity in tomatoes (Rodrigues and Furlong, 2022). Most of the studies evaluated postharvest application on strawberry fruits against fungal infections (Zatla et al., 2017; Wei et al., 2018; Abd-Elkader et al., 2021; de Oliveira Filho et al., 2021; Hassan et al., 2021). Preharvest application of tea tree oil gradually increased total phenolic content in strawberry fruits in the first days of storage (Wei et al., 2018). We registered gradually increasing antioxidant activity after preharvest treatment with coriander extract, however treatment with essential oil resulted in varied antioxidant capacity. There is a lack of information on how the preharvest application of plant products affects plants during growth. Our observations suggest an interesting hypothesis that secondary products like hydrosol could be useful additives to increase antifungal activity *in vivo*.

### 4.3 Photosynthetic response of strawberry leaves after application with coriander seed products

Stomatal conductance, transpiration rate and other photosynthetic capacity indices of plants may be affected by many environmental factors, like heavy metal pollution (Stancheva et al., 2014) or salt stress (Faghih et al., 2017). Coriander extract showed the potential to increase the photosynthetic capacity of infected strawberries. Similar data were obtained by (Yao et al., 2020), where seaweed extracts increased photosynthetic rate, chlorophyll content, and intercellular CO_2_ in tomatoes. The exception in our study was treatment with Ext 0.16+h, which did not increase photosynthetic rate that much. However, mostly increased stomatal conductance, transpiration rate and the ratio of intercellular CO_2_ (Ci/Ca) values. Essential oil, which in our case had the highest antifungal activity against grey mold, did not enhance the photosynthetic capacity of strawberries. Our study results provide no direct impact of increased photosynthetic capacity and antifungal effect after treatment with natural oils. It suggests that treatment with coriander extract may not be favorable for the plant, and its reaction was directed not to disease fighting but more to internal processes.

Clear differences after treatment with highest tested concentrations of essential oil and extract were indicated in our study. According to statistical analysis of strawberry’s physiological response to application of coriander products, inoculated plants and higher concentration (1.6%) coriander extract was grouped by similar plant response. Coriander extract in higher concentration caused plant stress similar to infection. Essential oils in high concentrations can have herbicidal activity, and completely kill plants (Tworkoski, 2002). Coriander essential oil increased photosynthetic capacity in low concentrations (0.12%) but with increased concentration (1.2%) photosynthetic rate and other photosynthetic parameters decreased lower compared to control plants. The herbicidal effect on the plant was possibly manifested under 1.2% of coriander essential oil and extract. High total phenolic compound contents and antioxidant scavenging activity can contribute to the statement.

## Data availability statement

The raw data supporting the conclusions of this article will be made available by the authors, without undue reservation.

## Author contributions

Conceptualization, LD, KL, AB, NR, and AVa; methodology, LD, KL, NR, and AVa; software, LD and KL; validation, AB and GS; performing the experiment, collecting the samples for analysis, RS, JM, KL, LD, and SC; formal analysis, LD and KL; investigation, AB, VV-K, NR, AVi, AVa, and GS; writing-original draft preparation, LD and KL; writing-review and editing, AB, NR, AVi, AVa, VV-K, and GS; visualization, LD and KL. All authors have read and agreed to the published version of the manuscript.
